# Supplementary material for: Vaccine-induced time- and age-dependent mucosal immunity to gastrointestinal parasite infection
Source: NPJ Vaccines. 2022 Jul 7;7:78. doi: 10.1038/s41541-022-00501-0 (PMC9262902; doi:10.1038/s41541-022-00501-0)
Supplement: Supplementary file 1 — Supplemental Material [file 41541_2022_501_MOESM1_ESM.pdf]

Vaccine-induced time- and age-dependent mucosal immunity to gastrointestinal parasite infection

Wei Liu[1,†], Tom N. McNeilly[2,†,*], Mairi Mitchell[2], Stewart T.G. Burgess[2], Alasdair J. Nisbet[2], Jacqueline B. Matthews[2,3,†], and Simon A. Babayan[1,2,†,*]

[1]Institute of Biodiversity, Animal Health and Comparative Medicine, University of Glasgow, Glasgow, G12 8QQ, Scotland, UK,
[2]The Moredun Research Institute, Pentlands Science Park, EH26 0PZ, Scotland, UK,
[3]Roslin Technologies Limited, Roslin Innovation Centre, University of Edinburgh, Easter Bush, EH25 9RG, Scotland, UK
[†]These authors contributed equally to this work
[*]Corresponding authors: tom.mcneilly@moredun.ac.uk, simon.babayan@glasgow.ac.uk

SUPPLEMENTARY INFORMATION

Supplementary Data 1

`multiqc-report-biopsy.html`: Quality check report of 180 RNA-seq sample FastQC reports using MultiQC[1].

Supplementary Data 2

`ipa_3V_6V.ods`: Ingenuity Pathway analysis (IPA) results for all the WGCNA clusters shown in Figure 2.

Supplementary Data 3

`gene-cluster-masigpro.ods`: Differentially expressed gene list between the age groups. The expression levels are shown in Supplementary Figure 5C.

Supplementary Figures

Study design

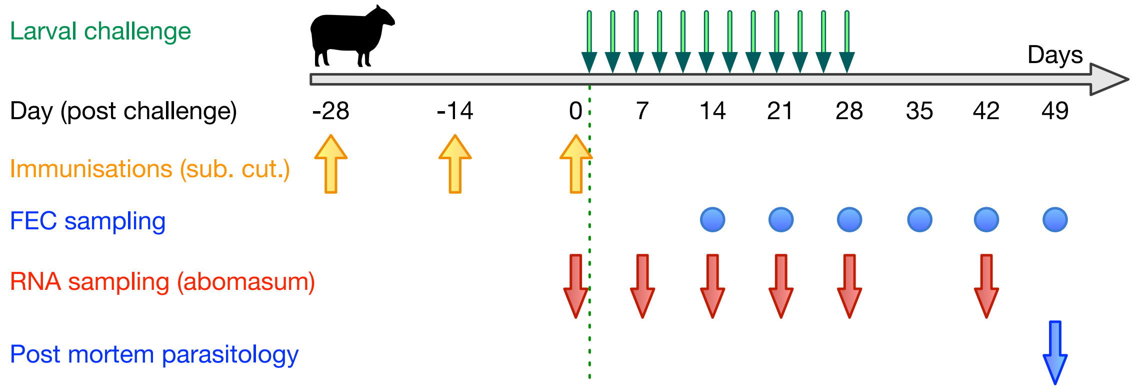

Supplementary Figure 1: Experimental design. Timeline of immunisation, challenge, and sampling of sheep.

Methods flow overview

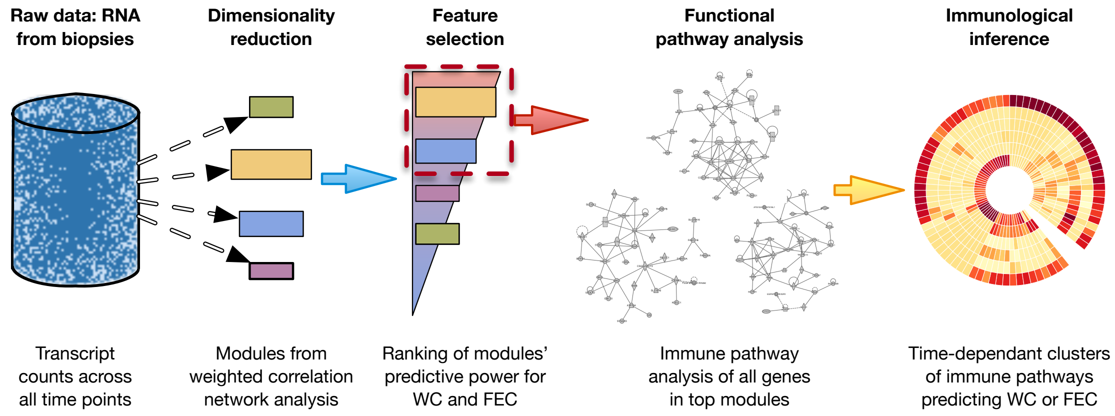

Supplementary Figure 2: Data analysis pipeline overview. Depiction of the flow through which raw transcript counts were taken to extract immunological information relevant to the response to vaccination and infection.

Clustering of all transcriptomes using t-SNE.

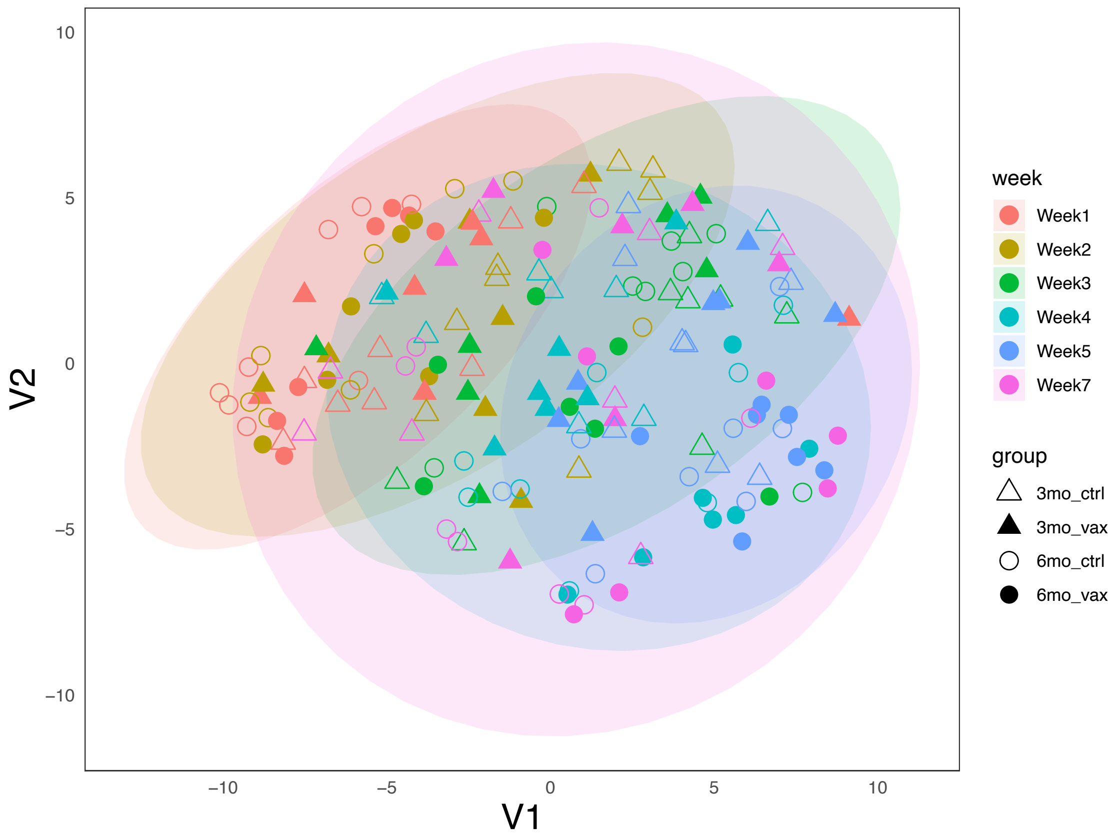

Supplementary Figure 3: Unsupervised clustering of transcriptomes per treatment and day post challenge. The ∼15K transcripts were reduced to two components using t-Distributed Stochastic Neighbour Embedding (t-SNE)[2] to visualise the datasets in two-dimensional space. Samples clustered weakly by treatment but more distinctly by day post challenge (DPC), specifically before *vs.* after 14 DPC.

Weight of WGCNA modules predicting parasite burdens.

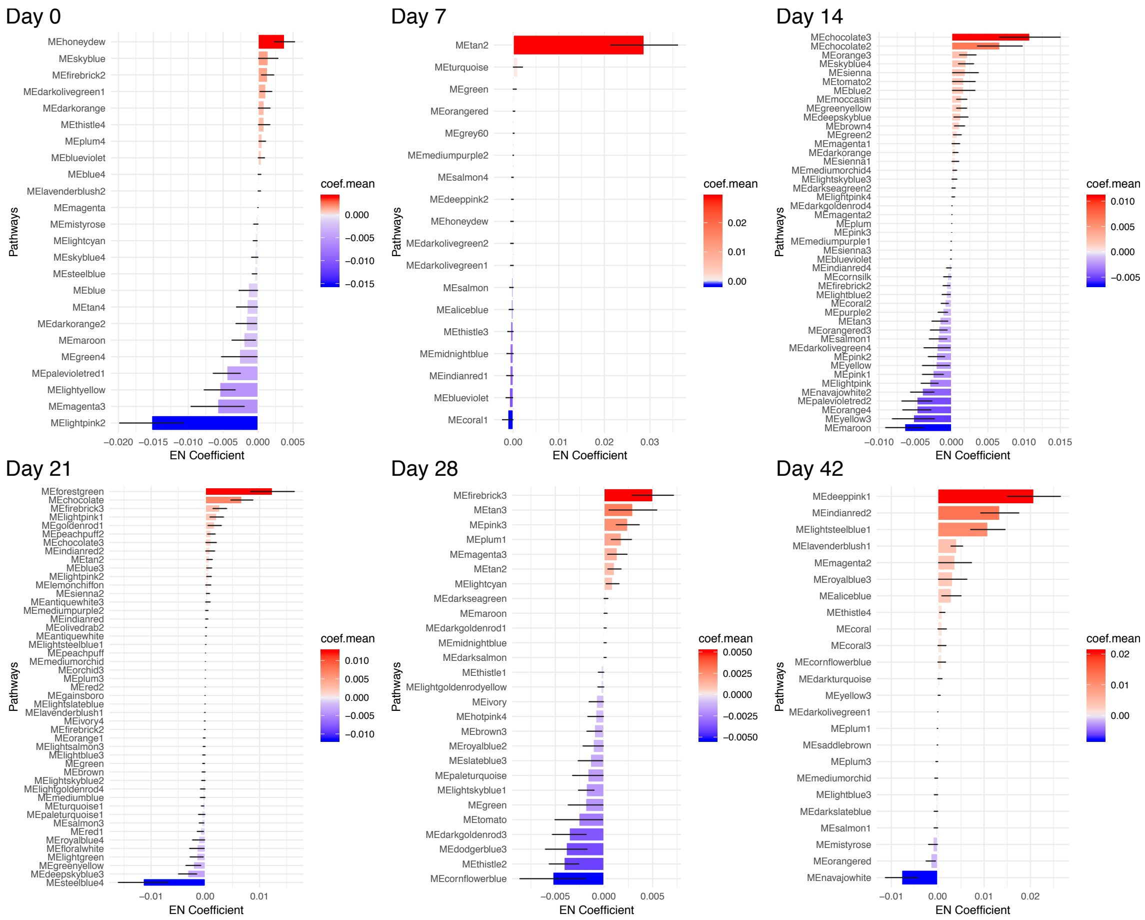

Supplementary Figure 4: ElasticNet coefficients bar plot of WGCNA modules associated with worm burden and cFEC across 42 days post challenge. Only modules with ElasticNet coefficients ≠ 0 are depicted and were retained for further analysis.

Differential expression analysis of genes included in WGCNA modules.

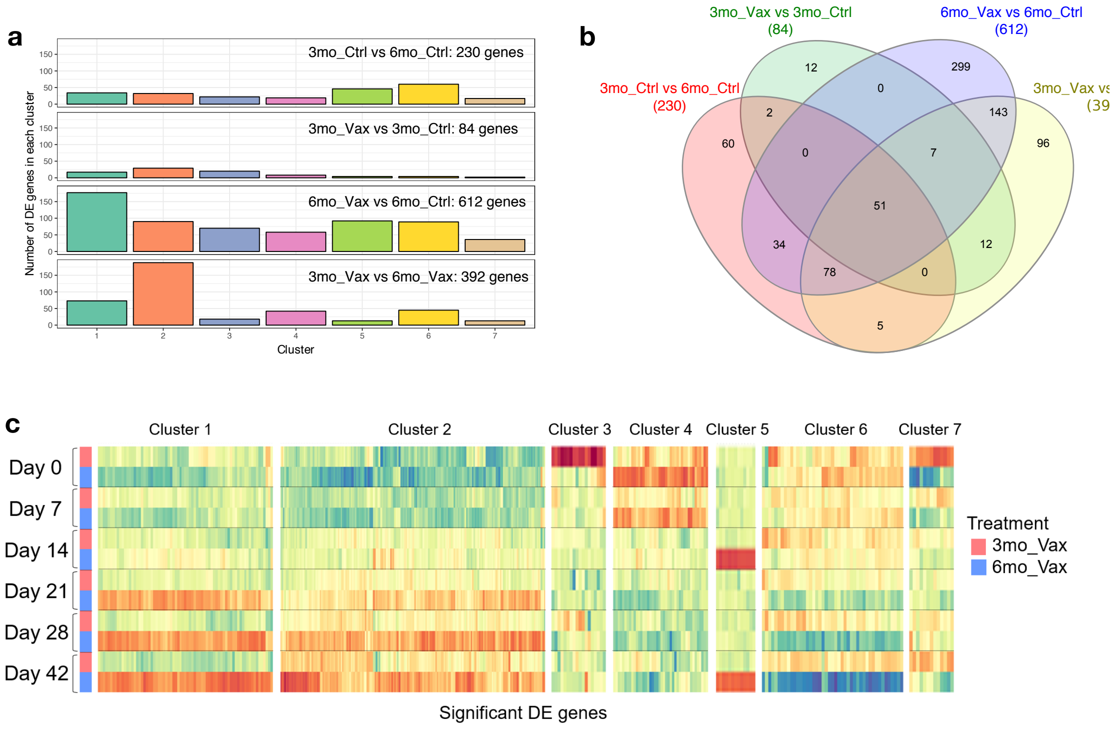

Supplementary Figure 5: Time dynamics of differentially expressed (DE) genes. a, Number of DE genes in each WGCNA modules within each of 4 pairwise DE pairwise comparisons: 3 month-old control *vs.* 6 month-old control ('3mo-Ctrl vs 6mo-Ctrl'), 3 month-old vaccinated *vs.* 3 month-old control ('3mo-Vax *vs* 3mo-Ctrl'), 6 month-old vaccinated *vs.* 6 month-old control ('6mo-Vax *vs* 6mo-Ctrl') and 3 month-old vaccinated *vs.* 6 month-old vaccinated ('3mo-Vax *vs* 6mo-Vax'). **b**, Venn diagram of all genes from four pairwise comparisons. **c** Heat map of the temporal expression patterns of significant DE gene expression. Individual genes within clusters 1—7 are shown in columns (see full gene list in Supplementary Data 2). Each row represents either 3mo or 6mo vaccinated lambs at different time points.

Time-course of immune pathways significantly represented in abomasal transcriptomes

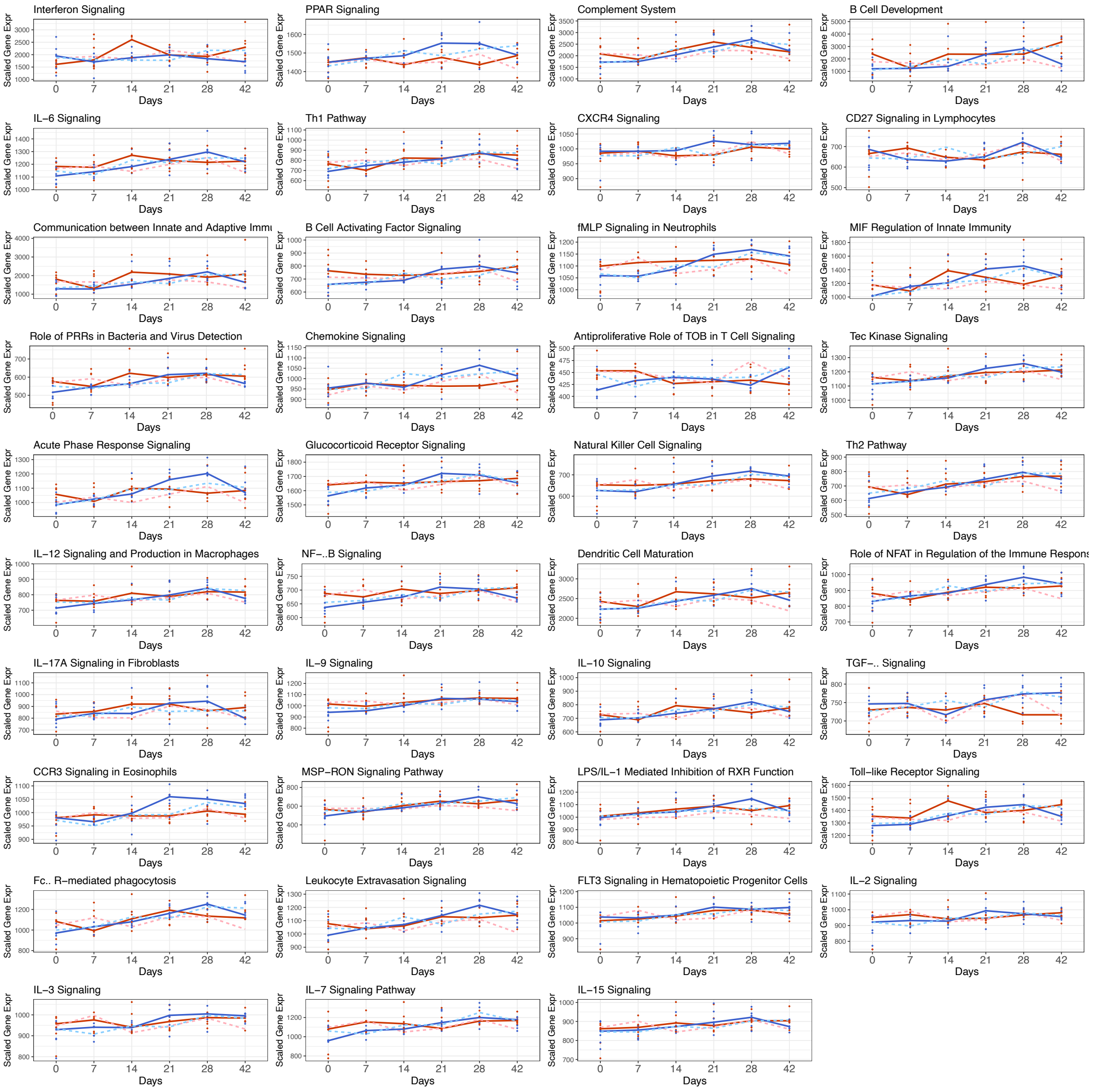

Supplementary Figure 6: Time course of pathways significantly represented in abomasal transcriptomes. Mean scaled expression levels in each pathway predictive of worm burdens and/or cFEC, selected in Figure 2. Points represent individual lambs at each time point and lines represent corresponding mean values for: vaccinated (solid lines); control (dashed lines); 3-month-old (red); and 6-month-old (blue) lambs.
