# Supplementary material for: Vaccine-induced time- and age-dependent mucosal immunity to gastrointestinal parasite infection
Source: NPJ Vaccines. 2022 Jul 7;7:78. doi: 10.1038/s41541-022-00501-0 (PMC9262902; doi:10.1038/s41541-022-00501-0)
Supplement: Supplementary file 2 — REPORTING SUMMARY [file 41541_2022_501_MOESM2_ESM.pdf]

nature portfolio

Corresponding author(s): Tom McNeilly
Simon Babayan

Last updated by author(s): Oct 22, 2021

Reporting Summary

Nature Portfolio wishes to improve the reproducibility of the work that we publish. This form provides structure for consistency and transparency in reporting. For further information on Nature Portfolio policies, see our Editorial Policies and the Editorial Policy Checklist.

Statistics

For all statistical analyses, confirm that the following items are present in the figure legend, table legend, main text, or Methods section.

n/a	Confirmed	
☐	☒	The exact sample size (*n*) for each experimental group/condition, given as a discrete number and unit of measurement
☐	☒	A statement on whether measurements were taken from distinct samples or whether the same sample was measured repeatedly
☐	☒	The statistical test(s) used AND whether they are one- or two-sided<br>*Only common tests should be described solely by name; describe more complex techniques in the Methods section.*
☐	☒	A description of all covariates tested
☐	☒	A description of any assumptions or corrections, such as tests of normality and adjustment for multiple comparisons
☐	☒	A full description of the statistical parameters including central tendency (e.g. means) or other basic estimates (e.g. regression coefficient) AND variation (e.g. standard deviation) or associated estimates of uncertainty (e.g. confidence intervals)
☒	☐	For null hypothesis testing, the test statistic (e.g. *F*, *t*, *r*) with confidence intervals, effect sizes, degrees of freedom and *P* value noted<br>*Give P values as exact values whenever suitable.*
☒	☐	For Bayesian analysis, information on the choice of priors and Markov chain Monte Carlo settings
☒	☐	For hierarchical and complex designs, identification of the appropriate level for tests and full reporting of outcomes
☒	☐	Estimates of effect sizes (e.g. Cohen's *d*, Pearson's *r*), indicating how they were calculated

Our web collection on statistics for biologists contains articles on many of the points above.

Software and code

Policy information about availability of computer code

Data collection	N/A
Data analysis	Bioinformatics were performed using Illumina CASAVA 1.8, FastQC 0.11.5, MultiQC, cutadapt 1.11, kallisto 0.46.2, and R v3.x packages tximport, DESeq2, maSigPro, and WGCNA. Machine learning and geenrlised linear modelling were performed in Python 3.6 using ElasticNetCV from ScikitLearn and statsmodels. Pathway analysis was performed using Ingenuity Pathway Anlaysis (Qiagen Inc.).<br>All code is available at https://github.com/SimonAB/Liu2021.

For manuscripts utilizing custom algorithms or software that are central to the research but not yet described in published literature, software must be made available to editors and reviewers. We strongly encourage code deposition in a community repository (e.g. GitHub). See the Nature Portfolio guidelines for submitting code & software for further information.

Data

Policy information about availability of data

All manuscripts must include a data availability statement. This statement should provide the following information, where applicable:

- Accession codes, unique identifiers, or web links for publicly available datasets
- A description of any restrictions on data availability
- For clinical datasets or third party data, please ensure that the statement adheres to our policy

Links to the raw data are all available at https://github.com/SimonAB/Liu2021

Field-specific reporting

Please select the one below that is the best fit for your research. If you are not sure, read the appropriate sections before making your selection.

☒ Life sciences ☐ Behavioural & social sciences ☐ Ecological, evolutionary & environmental sciences

For a reference copy of the document with all sections, see nature.com/documents/nr-reporting-summary-flat.pdf

Life sciences study design

All studies must disclose on these points even when the disclosure is negative.

Sample size	Experimental groups comprised vaccinated 3-month-old lambs (n = 15 [7 females, 8 males]), control (adjuvant only) 3-month-old lambs (n = 16 [8 females, 8 males]), vaccinated 6-month-old lambs (n = 15 [8 females, 7 males]), and control (adjuvant only) 6-month-old lambs (n = 16 [8 females, 8 males]). All lambs were infected with 2,000 T. circumcincta L3 stage larvae three times per week for four weeks beginning on the day of the final immunisation.
Data exclusions	Samples that were not part of this study were taken from the animals. No data that were intended for this study were excluded from the analysis.
Replication	A power analysis informed sample sizes for vaccine efficacy based on prior trials in the same system. Machine learning models were trained and validated on separate individuals to reduce overfitting and increase generalisability. However, the full experimental design was performed only once.
Randomization	Lambs were randomly allocated to four groups which were balanced for weight and sex.
Blinding	Blinding could not be formally conducted due to clearly visible procedures involving the experimental animals. However, parasite counting and processing of tissues for RNA extraction were performed blinded until complete. Data analysis was conducted independently by different team members who had group allocation information.

Reporting for specific materials, systems and methods

We require information from authors about some types of materials, experimental systems and methods used in many studies. Here, indicate whether each material, system or method listed is relevant to your study. If you are not sure if a list item applies to your research, read the appropriate section before selecting a response.

Materials & experimental systems

n/a	Involved in the study	
☒	☐	Antibodies
☒	☐	Eukaryotic cell lines
☒	☐	Palaeontology and archaeology
☐	☒	Animals and other organisms
☒	☐	Human research participants
☒	☐	Clinical data
☒	☐	Dual use research of concern

Methods

n/a	Involved in the study	
☒	☐	ChIP-seq
☒	☐	Flow cytometry
☒	☐	MRI-based neuroimaging

Animals and other organisms

Policy information about studies involving animals; ARRIVE guidelines recommended for reporting animal research

Laboratory animals	No laboratory animals were used.
Wild animals	No wild animals were used.
Field-collected samples	Farm sheep were used, all housed for the duration of the experiment in protected enclosures and monitored daily.
Ethics oversight	All animal procedures were performed at Moredun Research Institute (MRI) under Home Office licence 70/7914. Ethical approval was obtained from the MRI Animal Welfare and Ethical Review Body (E22/15).

Note that full information on the approval of the study protocol must also be provided in the manuscript.
